# Treatment of Hemorrhage

**Published:** 1853-04

**Authors:** F. L. Crane

**Affiliations:** Easton, Pennsylvania.


### ARTICLE VIII.

*Treatment of Hemorrhage.* By F. L. CRANE, D. D. S., Easton, Pennsylvania.

ON a leaf of my case book I find the following:—May 23d, 1851, 10 o'clock, A. M., extracted a tooth, the upper left second molar, for Mr. Stocker, of Mount Bethel, (in this county.) This gentleman has been under treatment for some time past for a gastrohepatic derangement, with a tendency to phthisis and a hemorrhagic diathesis. Informed me that he had had several attempts at extracting teeth, none of which succeeded. In this case the roots of the tooth were of extraordinary length. A small, thin piece of the alveolar plate came away with the tooth. The bleeding ceased, or nearly so, before he left the office, but he said soon commenced and bled throughout the day. In the evening I learned that he was inquiring for me somewhat anxiously. I found the gum bleeding profusely. Tried the usual styptics without much effect. Called Dr. V. M. Swayze, a skillful dentist, of this borough, to assist me. Plugged the cavity tightly with cotton, and placed a piece of cork upon it to shut against, tied a handkerchief over his head to keep his mouth shut, and dismissed him with little or no bleeding. In the night he awoke with his bed flooded with blood; one of our physicians, Dr. Abernethy, was sent for, who exhausted his skill in attempts to arrest the bleeding without effect. In the morning I was informed that Mr. Stocker was in danger of bleeding to death, and was requested to do what I could to prevent so dire a calamity. Dr. Abernethy proposed taking up the carotid artery. I told him I had yet one resource left, requesting him to do what he could for the gentleman, while I was making preparations. The patient had already fainted from the loss of blood, and the household were pretty thoroughly frightened, for a large quantity of blood had been lost. I went quickly to my office, and got wherewith to take an impression of the gum and remaining teeth, made a plaster cast, drying it over a spirit lamp; made metal casts and struck up a silver plate, soldered on clasps to fasten upon the remaining teeth, covered the plate with powdered alum—this last was perhaps of no use—had his mouth cleansed of coagulum, cotton, &c., and put the plate in its place; found the fit good, and the bleeding instantly ceased. Perhaps one circumstance which rendered my fixture successful was somewhat accidental. I found that one of my silver clasps was so broad that a lower tooth hit upon it when the mouth was closed, and pressed the plate tightly against the gum, rendering it impossible for the blood to escape. The plate was worn four days before removing it. Dr. Saltonstall's case, reported in the Journal of October last, differs somewhat from the above. Both methods had the same desirable results, viz. the salvation of life.

I was the more prompt in adopting the above plan to arrest hemorrhage in this case, from the fact that a child of a Mr. Randolph, in this place, had a short time previously died in consequence of continued hemorrhage from the gum. The child was teething; a physician lanced the gum, cutting down, as I understood, upon a lower incisor. The bleeding continued day after day, and the physicians were unable to arrest it. I heard of the case through one of the physicians, and began to cogitate upon what I would do in case I should be called upon, which I was not. I had resolved to strike up a plate, and make a soft pad to put upon it to close against the opposite gum, keeping the mouth closed by a bandage over the head, assisting the formation of coagulum by a styptic under the plate. Would such a proceeding answer, in the absence of so perfect a fixture as the "Alveolar Hemorrhage Compress" of Dr. Reid? The principal objection, perhaps, would be the necessity of removing it to give the child sustenance and then replacing it.

